# Dietary Exposure and Risk Assessment of Beta-Agonist Residues in Commercial Beef and Pork in Taiwan

**DOI:** 10.3390/foods12224052

**Published:** 2023-11-07

**Authors:** Shu-Han You, Chieh-Ning Lee

**Affiliations:** 1 Institute of Food Safety and Risk Management, National Taiwan Ocean University, Keelung City 20224, Taiwan; 2 Master Program of Food Safety Management, National Taiwan Ocean University, Keelung City 20224, Taiwan; 4094x005@gmail.com

**Keywords:** beef, pork, beta-agonists, dietary exposure, risk assessment

## Abstract

Beta-agonists (β-agonists) in meat products in one’s diet raise concerns about the possibility of foodborne illness. It may also lead to discomfort, such as headaches and occasional irregular heartbeats, which might be linked to a heightened concern for cardiovascular issues. Taiwan’s high demand for meat and reliance on imported meat products from certain countries where β-agonists are permitted has raised concerns. Recent import border checks and monitoring of meat products in the market have revealed the concentration of non-compliance with β-agonist residue regulations, which is ten ppb. This study aims to analyze the concentration of β-agonist residues in meat products sold in Taiwan and assess the current levels of exposure and dietary risk for consumers. The study analyzed 1415 samples of domestically produced and imported livestock products from supermarkets, traditional markets, and bulk stores in New Taipei City between 2019 and 2023. The samples were analyzed using the method for detecting 21 β-agonists based on the Taiwan Food and Drug Administration’s specifications. Estimated daily intake (EDI) of β-agonists for different age groups and the target hazard quotient (THQ) were used to assess dietary exposure and risk. The results showed that all 1415 samples were compliant with regulations. Among them, 43 beef samples showed residues of ractopamine originating from the United States, with residue concentrations ranging from 1 to 10 μg/kg and an average residue concentration of 3.3 ± 1.9 μg/kg. Under average consumption, the highest EDI for the exposed population was observed in the 6–12 age group, with values of 0.1469 μg/kg/day, 0.0734 μg/kg/day, and 0.0242 μg/kg/day for the three residue concentrations (maximum detected residue, maximum allowable residue, and average detected residue, respectively). The THQs for ractopamine in imported beef samples were all less than 1, indicating no health hazards at the current intake levels of each age group and the residue concentrations in commercially available beef. Despite the findings, traders need to acknowledge regulatory variations between Taiwan and exporting countries when importing meat products. Traders should provide inspection reports to monitor β-agonist residue levels in imports or explore sourcing beef from countries with β-agonist bans.

## 1. Introduction

Abuse of beta-agonists (β-agonists) as growth promoters has led to many problems, including poisoning of humans by ingesting meat containing β-agonist residues [1,2]. There were outbreaks of such food poisoning in Europe in 1990, in Shanghai, China, in 2006, and in Mexico in 2011–2013 [3,4,5]. When the human body ingests β-agonists, side effects can include headaches, rapid heartbeat, arrhythmia, vasodilation, muscle tremors, metabolic abnormalities, and reduced sensitivity to adrenergic receptors, and the risk of cardiovascular diseases such as hypertension, atherosclerosis, and heart disease may increase [6]. In addition, ingestion of β-agonists may cause hyperglycemia, hypokalemia, pulmonary edema, and myocardial ischemia [7].

Initially, β-agonists were used in the treatment of human and animal diseases, such as asthma and shock, as well as the prevention of miscarriage [8]. Fenoterol, isoxsuprine, and ritodrine can be used as short-acting miscarriage prevention (preventing preterm labor and miscarriage) drugs for human beings by inhibiting the contraction of smooth muscle and preventing the uterus from over-contracting, which may lead to fetal instability or preterm labor [7]. Salmeterol and Formoterol are long-acting β-sympathomimetic agents used in humans for the treatment of asthma or chronic obstructive pulmonary disease [9]. As animals age, their muscle-building capacity decreases. Thus, in order to promote muscle growth and improve feed utilization efficiency, livestock farmers usually feed feeds supplemented with β-agonists 20 to 40 days before slaughter [10].

Meat is one of the main sources of protein for humans today. According to a research survey by González et al. (2020) [11], by 2016, global meat intake had reached 500 times the amount it was in 1992, and this trend continues to grow. According to the 2021 trade statistics released by the Minister of Council of Agriculture of Taiwan, regarding meat, the top three major beef exporters to Taiwan are the United States (126,000 tons, 43.9%), Paraguay (72,700 tons, 25.3%), and Australia (44,900 tons, 15.7%); and the top three for pork are Spain (88,000 tons, 48.8%), Canada (37,200 tons, 20.6%), and Denmark (27,500 tons, 15.3%) [12]. In terms of offal, the major beef offal exporters to Taiwan are Paraguay (7200 tons, 34.9%), New Zealand (5000 tons, 24.0%), and Australia (3900 tons, 18.9%), while the major pork offal exporters to Taiwan are Canada (12,700 tons, 32.9%), the Netherlands (6600 tons, 17.2%), and Spain (6500 tons, 16.9%) [12].

The Taiwan Ministry of Health and Welfare conducts border inspections on beef and pork imported from the United States. In 2021, a total of 476 batches of beef were sampled and tested, among which 473 batches met the standards, 190 batches were found to contain ractopamine residues (concentration between 1–10 μg/kg), and one batch was substandard (residue concentration of 20 μg/kg) [13]. Meanwhile, 15 batches of beef offal were tested, all of which met the standards. Among them, three batches were found to contain ractopamine residues (concentration between 1–2 μg/kg) [13]. In addition, 131 batches of pork and 132 batches of pork offal were tested, and no ractopamine residue was detected. Information on non-compliant foods from border inspection by the Taiwan Food and Drug Administration indicates that one batch of Honduran beef and two batches of Nicaraguan beef showed residues of 2 μg/kg zilpaterol in 2021, and one batch of Nicaraguan beef was detected with residues of 3 μg/kg zilpaterol in 2023 [13].

Every year, the US Food Safety and Inspection Service (FSIS) detects 1–3 cases of illegal use of salbutamol in meat products including beef, beef kidney, and beef liver by livestock farmers in the United States [14]. The domestic meat surveillance results show that ractopamine residues in beef liver exceed the standard, and livestock farmers in the United Stated may be involved in serious violations of the law on the use of salbutamol. Various countries have inspected the residue situations of β-agonists in meat products such as beef liver, pork liver, and chicken liver, with clenbuterol, ractopamine, and salbutamol being the most commonly detected. Meanwhile, the types of meat found to contain β-agonists are mainly beef (including offal) and pork (including offal) [2,3,15,16].

For the detection of β-agonists, the Taiwan Food and Drug Administration announced the “Method of Test for Veterinary Drug Residues in Foods—Test of Multiresidue Analysis of β-Agonists (MOHWV0041)”, which involves the detection of zilpaterol. Commonly, the hydrolysis time for sample pretreatment and instrumental analysis time may affect the test efficiency [17]. The method announced by the Taiwan Food and Drug Administration addresses this problem and allows test results to be obtained more quickly, thereby being appropriate for detecting the possible residues of β-agonists in imported meat.

Residues of β-agonists in meat products have been a concern since 2006, and ractopamine residues have been allowed in imported beef since 2012 and in pork (including offal and fat) since 2021. Since residues were allowed in imported meat, as revealed by border inspection of imported meat and sampling of meat for sale in the market, some imported meat products were still detected to be substandard in terms of β-agonists. Taiwan’s beef imports have been increasing year by year, and some beef- and pork-exporting countries, such as the United States, Canada, and Australia, allow residues of β-agonists, including ractopamine and zilpaterol [18,19,20]. Meat consumption in Taiwan has been increasing over the years, and increased exposure to β-agonist residues in meat may pose health risks. The objectives of this study are to analyze the concentration of β-agonist residues in meat products sold in Taiwan and to assess the current levels of exposure and dietary risk for consumers.

## 2. Materials and Methods

### 2.1. Data Analysis

This study was authorized by the Department of Health, New Taipei City Government, to use the sampling data from the Post-market Surveillance of Foods between 2019 and April 2023 as the sample for analysis. The samples were collected by health inspectors of the Department of Health from wholesale markets, importers, food manufacturers, large and medium-sized merchants, traditional markets, specialty restaurants, large supermarket chains, and mass merchandisers. The origins of the meat or the importers were distributed across the northern, central, and southern counties and cities. Samples of beef and pork (including offal and fat) were categorized into domestic and imported according to their sources. Among the total of 1415 samples collected, domestic samples accounted for 32 samples of beef (including 1 beef tripe sample) and 947 samples of pork (including 7 pork liver samples, 1 pork pancreas sample, 1 pork tripe sample, 9 pork intestine samples, and 1 pork heart sample), while imported samples were 206 samples of beef (including 1 beef liver sample, 3 beef tripe samples, and 1 beef heart sample) and 230 samples of pork (including 16 pig intestine samples, as shown in Table 1.

### 2.2. β-Agonists Analysis

Considering the validity and stability of the test method and its ability to detect residues of 21 β-agonists in meat, the Taiwan Food and Drug Administration announced the “Method of Test for Veterinary Drug Residues in Foods-Test of Multiresidue Analysis of β-Agonists (MOHWV0041)” (Taiwan Food and Drug Administration, 2019–2021). This method was chosen to test the muscle, offal, and fat samples from 2019–2023 involved in this study. Typically, the beef (including muscle, offal, and fat) or pork (including muscle, offal, and fat) sample was finely chopped and homogenized, and 2 g of the sample was weighed finely and placed in a 50-mL centrifuge tube. Subsequently, 20 μL of mixed internal standard solution at a concentration of 1 μg/mL (the concentration of internal standard in the final solution is equivalent to 10 ng/mL), 15 mL of 0.2 M sodium acetate buffer solution, and one ceramic homogenizer were added. Extraction was then performed using a homogenizer at 1000 rpm for 10 min. Afterwards, 100 µL of β-glucuronidase solution was added to the mixture and mixed well, followed by hydrolysis in a 37 °C water bath for 1 h. Then, 2 mL of concentrated hydrochloric acid was added and the mixture was shaken for 10 min. Subsequently, centrifugation was performed at 10,000 rpm for 10 min at 4 °C, the supernatant was extracted into a 15-mL centrifuge tube and then centrifuged at 3500 rpm for another 10 min. The final supernatant was used for purification.

The supernatant was injected into a solid-phase extraction cartridge (Bond Elute Plex PCX, 200 mg, 6 mL) that had been pre-washed with 6 mL of methanol and 6 mL of deionized water, and the effluent was discarded. The cartridge was then washed with 12 mL of 0.2 M hydrochloric acid, 12 mL deionized water, and 12 mL methanol, in turn. The effluent was discarded. The extract was rinsed with 12 mL methanol: ammonia (95:5, *v*/*v*) solution, and the rinsate was collected and dried under nitrogen flow at 65 °C. The residue was added to 1 mL of 5 mM ammonium acetate:methanol (9:1, *v*/*v*) solution and subjected to oscillatory dissolution using a vortex mixer to be used as the stock solution for analysis. To prepare the test solution, 500 μL of stock solution was mixed with 500 μL of 5 mM ammonium acetate:methanol (9:1, *v*/*v*) solution and filtered through a 0.22-μm Nylon filter membrane. The chromatographic column was ZORBAX RRHD Eclipse Plus C18, 3 × 150 mm, 1.8 µm, and the analysis was carried out on a liquid chromatography tandem mass spectrometer (Agilent 1290 Infinity II + 6495 Triple Quad LC/MS, 6495 Triple Quadrupole LC/MS System, G6495BA, Agilent, Santa Clara, CA, USA). The chromatographic and analytical conditions are as shown in Table S1.

Considering the test efficiency and to simultaneously detect residues of 21 β-agonists in meat, the Taiwan Food and Drug Administration publicly amended the test method and announced the “Method of Test for Veterinary Drug Residues in Foods—Fast Extraction Method for Multiresidue Analysis of β-Agonists” [21]. This method was used to analyze muscle samples from May to October 2021. Typically, the beef or pork muscle sample was finely chopped and homogenized, and 2 g of the sample was weighed finely and placed in a 50-mL centrifuge tube. Subsequently, 20 μL of mixed internal standard solution at a concentration of 1 μg/mL (the concentration of internal standard in the final solution is equivalent to 10 ng/mL), 1 mL of 0.05 M hydrochloric acid solution, and one ceramic homogenizer were added. The mixture was subjected to oscillation extraction with a homogenizer at 1000 rpm for 5 min. Afterwards, 10 mL of 1% acetonitrile acetate solution was added, and oscillation extraction was performed at 1000 rpm for 5 min, followed by centrifugation at 4000× *g* for 5 min at 4 °C.

Then, 5 mL of the supernatant was injected into the fast-extraction cartridge of β-agonists (FaPEx-VAG), and the flow rate was controlled at 1 drop/second. The effluent was collected to be used as the stock solution for tests. As for the preparation of the test solution, 2.5 mL of the stock solution was dried under nitrogen flow at 65 °C, and the residue was dissolved with 1 mL of 0.1% formic acid:methanol (9:1, *v*/*v*) solution, mixed homogeneously, and then filtered through a 0.22-μm Nylon filter membrane. The chromatographic column was ZORBAX RRHD Eclipse Plus C18, 3 × 150 mm, 1.8 µm; analysis was performed on a liquid chromatography tandem mass spectrometer (Agilent 1290 Infinity II + 6495 Triple Quad LC/MS, 6495 Triple Quadrupole LC/MS System. G6495BA, Agilent, Santa Clara, CA, USA), and the chromatographic and analytical conditions are as shown in Table S1.

### 2.3. Dietary Exposure and Risk Estimate

**Figure 1:**
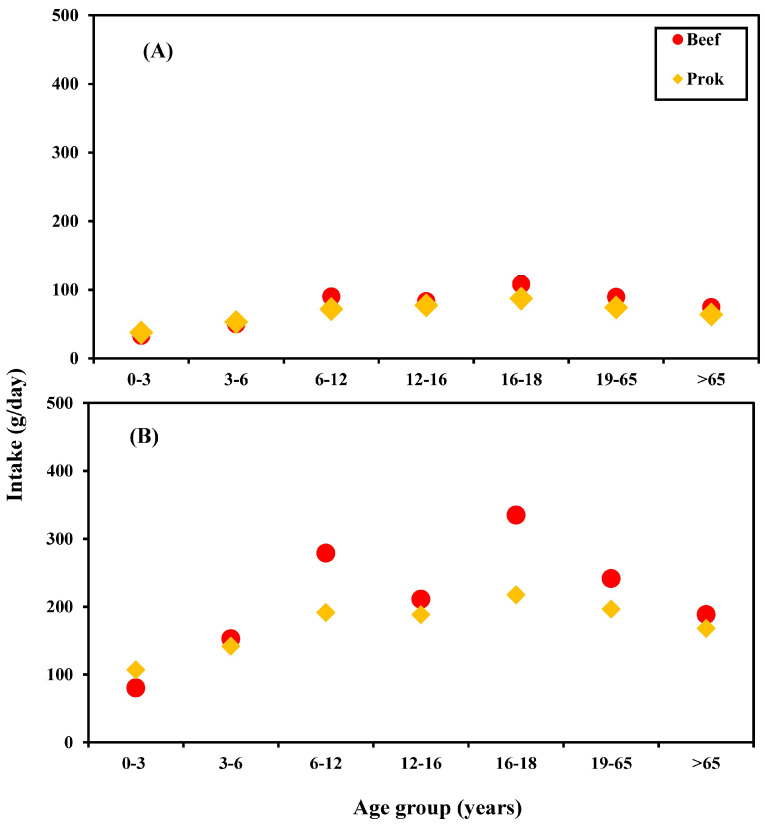
(**A**) Average and (**B**) 95th percentile meat intake (g/day) of each age group in 2017–2020 [22].

In this study, we refer to Chaleshtori et al. (2018) to determine the estimated daily intake (EDI) of meat containing β-agonists of each age group, and the formula is EDI = ∑(*DI* ÷ 1000 × *R* × *C*) ÷ BW. In the formula, *DI* is the average daily intake of meat (g/day) or the 95th percentile intake (g/day) of each age group, which was determined referring to 2017–2020 statistics on daily meat intake in the Taiwan National Food Consumption Database [22], as shown in Figure 1 and Table S2; *R* is the proportion of each imported meat product, which was estimated referring to the concept of Rustia et al. (2022) [23] combined with the 2020 Annual Report on Supply and Demand for Food released by the Minister of Council of Agriculture of Taiwan [24]. Accordingly, the proportions of beef and pork imported (imported quantity divided by domestic supply) were calculated to be 96.1% and 9.2%, respectively; *C* represents the concentration of β-agonist residues in meat (ug/kg). In this study, three residual concentrations were used for exposure assessment: the maximum concentration of β-agonist residues detected in imported meat (the maximum concentration of ractopamine residue refers to the concentration of 20 μg/kg detected in beef at border inspections in 2021) [13], the maximum residue limit (MRL) [25] and average residual concentration of residues in meat products; and BW is the average body weight (kg) of each age group [22].

Referring to [26], this study estimated the target hazard quotient (THQ) of each age group to assess the potential health risk of consuming meat products containing β-agonists. The formula is THQ = EDI ÷ ADI, where ADI is the acceptable daily intake of β-agonists established by JECFA, e.g., 1 µg/kg bw/day for ractopamine, 0.004 µg/kg bw/day for clenbuterol, and 0.04 µg/kg bw/day for zilpaterol [27]. A higher THQ means a greater risk, and a THQ greater than 1 indicates a non-carcinogenic health risk [26].

## 3. Result

### 3.1. Concentrations of Ractopamine Residues in Commercially Available Meat Products

Table 2 lists the number of domestic and imported samples of commercially available beef and pork (including offal and fat) detected in 2019–2023. The total number of imported beef samples for sale was 206 (Table 1). The countries of origin for imported beef samples include the United States, Australia, New Zealand, Paraguay, Japan, Panama, Honduras, the Netherlands, and Nicaragua. The countries of origin for imported pork samples include the United States, Spain, Canada, the United Kingdom, Denmark, the Netherlands, France, and Italy. Among the 43 samples that showed β-agonist residues, all were imported from the United States, with the β-agonist residue being ractopamine in all cases. No β-agonists were detected in domestic beef samples or domestic and imported pork samples sold in the market.

**Figure 2:**
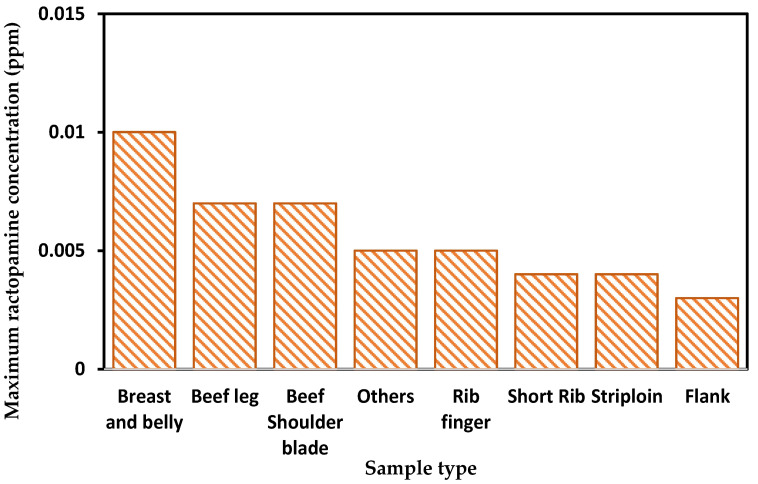
The relationship between maximum ractopamine concentration and sample type.

Table 3 shows the ractopamine residue concentrations and positivity rate of imported samples of commercially available beef in 2019–2023. The ractopamine residues in imported beef samples from 2019 to 2023 reveal a persistent presence. The concentration of ractopamine residues in imported beef was 4.0 ± 1.4 (mean ± standard deviation). The wide variability in the positivity rate of ractopamine in imported beef ranged from 9.5–32.0% (min–max) in 2019–2023. Among the sample types of detected beef, breast and belly meat, beef shank, and shoulder blades contained the highest levels of ractopamine (Figure 2).

### 3.2. Dietary Exposure to Ractopamine in Imported Commercially Available Beef

The EDI (µg/kg/day) of ractopamine from imported beef of each age group in terms of average and 95th percentile of intake. Regarding the average intake, the top three age groups in terms of the highest EDIs are 0–3 years, 3–6 years, and 6–12 years. Specifically, the three residue concentrations (max concentration of 20 μg/kg, MRL of 10 μg/kg, and av. concentration of 3.3 μg/kg) for the three age groups are 0.0491, 0.0245, and 0.0081 μg/kg/day for children aged 0–3 years, 0.0477, 0.0239, and 0.0081 μg/kg/day for children aged 3–6 years, and 0.0473, 0.0237, and 0.0078 μg/kg/day for children aged 6–12 years (Table S3). In terms of the 95th percentile, the top three age groups in terms of the highest EDIs regarding the three residue concentrations are children aged 6–12 years (0.1469, 0.0734, and 0.0242 μg/kg/day), children aged 3–6 years (0.1466, 0.0733, and 0.0242 μg/kg/day), and children aged 0–3 years (0.1202, 0.0601, and 0.0198 μg/kg/day) (Table S4).

### 3.3. Health Risk Evaluation

**Figure 3:**
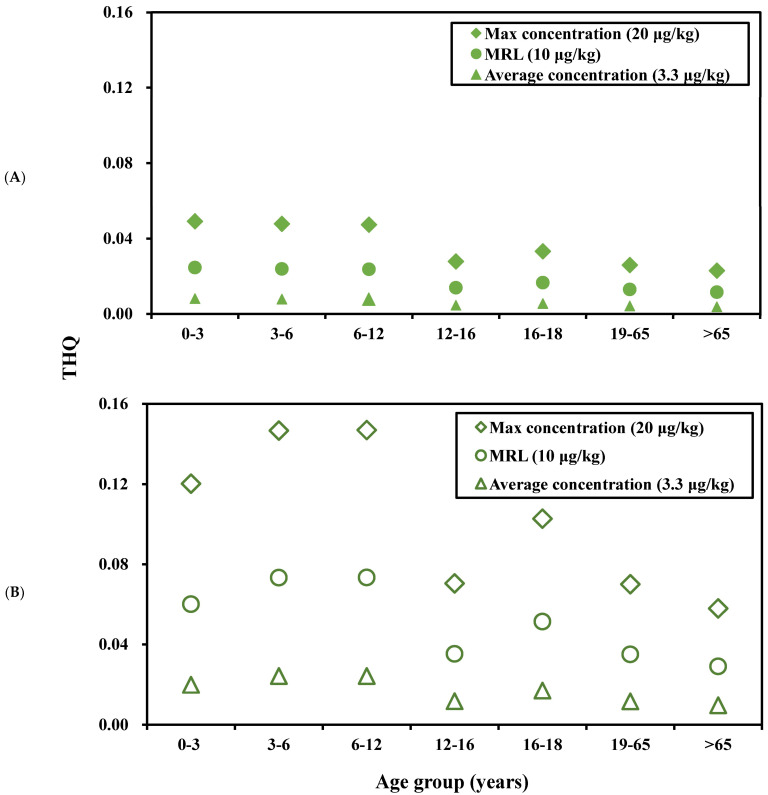
Target hazard quotient (THQ) of ractopamine from imported beef of each age group based on (**A**) average and (**B**) 95th percentile of intake and residue concentrations. (EDI is the estimated daily intake; MRL is the maximum residue limit).

Figure 3 shows the target hazard quotient (THQ) of ractopamine in beef of each age group in terms of the average and 95th percentile intakes. With regard to the average intake, the top three age groups in terms of highest THQs regarding the three residue concentrations are children aged 0–3 years (0.0491, 0.0245, and 0.0081), children aged 3–6 years (0.0477, 0.0239, and 0.0079), and children aged 6–12 years (0.0473, 0.0237, and 0.0078) (Figure 3A). The data details are presented in Table S3.

In terms of the 95th percentile, the top three age groups in terms of highest THQs regarding the three residue concentrations are children aged 6–12 years (0.1469, 0.0734, and 0.0242), children aged 3–6 years (0.1466, 0.0733, and 0.0242), and children aged 0–3 years (0.1202, 0.0601, and 0.0198) (Figure 3B). The detailed data are listed in Table S4.

## 4. Discussion

### 4.1. Ractopamine in Imported Beef

This study analyzes the sampling inspection results of commercially available beef in Taiwan in 2019–2023. The residue of β-agonist ractopamine was detected only in beef samples imported from the United States. Meanwhile, β-agonist residues were not detected in imported or domestically-produced pork. This study is based on the Post-market Surveillance of Foods of the Department of Health, New Taipei City. The sampling data include beef and pork (including offal and fat) meat products, with 111 batches of imported beef sampled for inspection, of which 43 were found to contain ractopamine residues. In Taiwan, 96.1% of beef (including offal) products are imported. The United States is the main source (including offal), and the annual import volume continues to grow, which is probably due to the stable supply of beef from the United States and its large production volume (12.733 million tons in 2020). The present findings suggest that traders should recognize the difference in the regulations and limits between Taiwan and the exporter when importing meat products. Traders should be required to issue relevant inspection reports to control the amount of β-agonist residues in imported meat products, or consider importing beef from countries that prohibit the use of β-agonists, such as New Zealand, which is in the scope of the approved importation of beef products (β-agonist residues are prohibited in beef).

In this study, no β-agonists were detected in imported pork (including offal) samples. The main countries Taiwan imports pork (including offal) from include Spain, Canada, Denmark, and the Netherlands. Among them, Spain, Denmark, and the Netherlands are members of the European Union, which prohibits the use and residue of β-agonists in animals [28]. Canada is the world’s third largest pork exporter. Because some pork trade markets do not allow ractopamine residues, to maintain economic trade, the Canadian Pork Board has indicated that all pork products exported from Canada are free of ractopamine residues [29].

In the 2019–2023 samples in this study, ractopamine was only detected in the muscle samples among imported commercially available beef samples, and the residue concentration ranged from 1–10 μg/kg (min–max), with average residue concentration of 3.3 ± 1.9 μg/kg. The concentrations of ractopamine in all samples are lower than the MRL of ractopamine (10 μg/kg) in imported beef. Sung et al. (2015) [30] examined ractopamine residues in 299 imported meat products (154 beef, 88 processed beef products, and 57 pork), and ractopamine residues were detected in two beef samples at concentrations of 0.6 and 2.0 μg/kg, respectively. Mostafa et al. (2022) [31] examined ractopamine residues in 21 imported meat products sold in Egypt, among which 10 (including beef, pork, and processed meat) had ractopamine residues at concentrations ranging from 1.14 to 6.30 μg/kg, in compliance with the JECFA standard (10 μg/kg). These results are consistent with the present study, in which, although ractopamine residues were frequently detected in imported meat, the residue concentrations were less than 10 μg/kg.

In 2018–2022, the US FSIS investigated β-agonist residues in commercially available beef, beef liver, pork, and pork liver. Ractopamine was detected in beef, beef liver, and pork liver, with residue concentrations being 3.2–11.9, 24.0–247.6, and 17.8–105.5 μg/kg, respectively. Offal (e.g., beef liver and pork liver) is more likely to retain ractopamine than muscle, which is consistent with the findings of Pleadin et al. (2012) [32]. After feeding pigs with 9 mg/kg ractopamine daily for 28 days, no ractopamine was detected in muscle the next day after drug withdrawal, whereas at least 8 days was required after drug withdraw for the offal to be free from ractopamine.

The dietary habits in Taiwan are influenced by the Chinese culture, and most people consume livestock offal. In Taiwan, pork offal is mainly imported from the United States, Canada, and the Netherlands, and beef offal is mainly imported from Australia, New Zealand, and Nicaragua. Among them, compared to the MRL set in Taiwan, the United States and Canada have higher MRLs for ractopamine residues in pork liver, and Australia has higher MRLs for ractopamine residues in beef offal (kidney and liver). Therefore, it is recommended to increase the number of offal samples in the future. In this study, only beef and pork samples were analyzed, but it needs to be noted that ractopamine and salbutamol have been previously detected in poultry products in Taiwan. Therefore, it is recommended that post-market surveillance should analyze more poultry samples. In addition, this study only analyzes the beef and pork samples collected in New Taipei City, and it is recommended to analyze the exposure to β-agonist residues in meat products and the associated health risks in each county and city.

### 4.2. Ingestion Exposure Risks

In this study, β-agonists were not detected in the samples of domestic beef (including offal), domestic pork (including offal), or imported pork (including offal); thus, there is probably no ingestion risk from these meat products. The risk assessment results of ractopamine in imported beef showed that in terms of the average and 95th percentile intakes, the THQs of ractopamine in imported beef were less than 1 regarding all three residue concentrations, indicating no immediate hazard from current daily intake of meat and the amount of ractopamine residue. For example, for children aged 6–12 years with a residue concentration of 20 μg/kg at the 95th percentile intake, the daily beef intake would need to be at least seven times the current intake (279.07 g/kg) or the residue concentration of ractopamine to be increased to 140 μg/kg in order for the THQ to be greater than 1. Therefore, there is no health concern regarding β-agonist residues in the current meat consumption environment in Taiwan, and the MRLs set by the government are in line with the current consumption environment.

JECFA has only established ADI for three β-agonists: ractopamine 1 µg/kg bw/day, clenbuterol 0.004 g/kg bw/day, and zilpaterol 0.04 µg/kg bw/day [27]. The ADIs of β-agonists in the United States are 1.25 and 0.083 μg/kg bw/day for ractopamine and zilpaterol [33], respectively, while those in Australia are 1 and 0.04 μg/kg bw/day [34], respectively. In Japan, the ADIs of β-agonists are 1, 0.004, and 0.083 μg/kg bw/day for ractopamine, clenbuterol and zilpaterol, respectively [35,36,37]. The Taiwan Food and Drug Administration has set the ADI for ractopamine at 1 μg/kg bw/day.

According to the JECFA study, in the clinical trial of ractopamine, six adult male participants (average body weight of 75 kg and average age of 23.5 years) were given oral doses of ractopamine at 5, 10, 15, 25, and 40 mg. Among participants who received doses of 10 mg or more, responses including cardiac acceleration, increased cardiac output, shortened cardiac cycle, and increased systolic blood pressure were observed. JECFA determined the No Observed Effect Level (NOEL) for ractopamine to be 5 mg, which was obtained by dividing the NOEL of 67 μg/kg bw/day by a safety factor of 50 [38] (taking into account a 10-fold for individual variation and a 5-fold for susceptibility and the small sample size). However, the Panel on Additives and Products or Substances used in Animal Feed considered the JECFA study as not sufficient to justify a NOEL of 5 mg for ractopamine and that the safety factor did not sufficiently take into account the possibility that high-risk groups may experience discomfort, even if the symptoms are short-lived. For example, newborns (i.e., under 18 months) may not be able to fully metabolize and inactivate the drug due to poor hepatic glucuronidation capacity, and people with cardiovascular disease and children with low tolerance to β-agonists are high-risk groups [28].

### 4.3. Administration of β-Agonists

This study compiles information on MRLs of β-agonists in meat that have been established by different countries and international organizations. Countries such as Russia and China, as well as country groups such as the European Union, have banned the use of β-agonists and banned the importation of meat containing β-agonist residues [28,39,40]. Some β-agonists, such as ractopamine and zilpaterol, are currently accepted as feed additives in some countries, including the United States and Canada, that allow the use of ractopamine in pigs, cattle, and turkeys [18,20]. In Taiwan, β-agonists are completely banned in domestic poultry and livestock farming [25].

Internationally, MRLs for ractopamine are widely determined, while only a few countries and international organizations, such as the United States, Japan, South Korea, and JECFA, have established MRLs for zilpaterol in beef. Regions and international organizations that have established MRLs for ractopamine in beef are Australia, Taiwan (for imported beef only), the United States, JECFA, Vietnam, Ghana, Japan (for imported beef only), and South Korea. Specifically, MRLs in beef muscle and beef liver range from 10–30 µg/kg and 40–90 µg/kg, respectively. While Taiwan and the United States do not have MRLs in beef kidney, all the other countries have set a 90 µg/kg MRL of ractopamine in beef kidney. Regions and international organizations that have set MRLs for ractopamine in pork are New Zealand, Brazil, Malaysia, Taiwan (for imported pork only), Canada, the United States, JECFA, Vietnam, Ghana, Japan (for imported pork only), and South Korea. The ranges of MRL in pork muscle and pork liver are 10–50 µg/kg and 40–150 µg/kg, respectively. The United States does not have a MRL set for ractopamine in pork kidney, while the MRLs in pork kidney set by the other countries range from 40–90 µg/kg.

However, due to the demand of international trade and to maintain the economic value of domestic meat products, Taiwan has only established MRL for ractopamine in imported meat. With reference to the international management of ractopamine, the risk analysis of food safety, and the MRL standard for ractopamine set by JECFA, and considering that the dietary habits of people in Taiwan include consumption of offal, the MRLs of ractopamine in imported beef and pork set in Taiwan are the minimum values of the internationally acceptable limits of β-agonist residues [41,42].

The United States has a sampling and monitoring program for domestic and imported meat, poultry, and egg products. The United States National Residue Program (NRP) is an interagency annual program in which the US FSIS, Food and Drug Administration, Environmental Protection Agency, and Department of Agriculture discuss and formulate the chemicals and items to be monitored and tested. Domestic meat products are directly sampled at slaughterhouses, while imported meat products are sampled at customs. Sampling methods include random sampling, enhanced sampling, and strengthened sampling, with the latter being conducted for manufacturers that have failed in the past [14].

According to the 2018–2019 NRP Blue Book (Residue Sampling Plan), the first phase of sampling is conducted annually for products with more than 95% consumption domestically, and the samples cover mainly livestock meat (beef, pork, lamb, and poultry), dairy products, and aquatic products. The FSIS determines the sample size on a table relating sample size to violation rate. For example, if the predicted violation rate is less than 0.29%, a minimum of 793 samples are required to meet the 90th percentile confidence interval. Therefore, FSIS has set a target of around 800 samples of each item per year, making an annual total of 7000 to 8000 samples. The samples will be examined by FSIS and the data will be reported to the Residue Violator Tracking System (RVT), and the Food and Drug Administration will conduct relevant statutory determinations and notify the relevant traders to improve the quality, recall the products, and remove the products from the shelves [14].

In Taiwan, the sales channels of domestic and imported meat products are widely distributed. Beef and pork are mainly produced in the south-central part of Taiwan. Beef is generally sold directly from slaughterhouses to regional restaurants or traditional markets, and pork is mainly traded to counties and cities through regional meat markets. Imported beef is mainly supplied to restaurants and mass merchandisers. Imported food monitoring in Taiwan can be divided into two stages. The first is border inspection, including batch-by-batch inspection, sampling inspection, batch-by-batch verification, or certification inspection conducted according to the Regulations of Inspection of Imported Foods and Related Products [43]. The number of inspections and sample size are determined referring to the Key Point Schedule of Regulations of Inspection of Imported Foods and Related Products [44]. The second stage is domestic distribution, in which regulation is carried out via post-market surveillance.

Since 2004, the Taiwan Food and Drug Administration has formulated an annual Post-market Surveillance of Foods for drug residues in livestock and aquatic products (hereinafter referred to as Post-market Surveillance of Foods). The surverillance mainly aimed at, through sampling and testing, commercially available poultry and aquatic products with a high failure rate in the past, with high public consumption volume (e.g., pork), involved in issues of concern to consumers (e.g., imported pork and beef), and with potential risks [45].

On-site inspection is conducted at food manufacturers or vendors by health inspectors from the Department of Health of each county and city in accordance with “The Regulations on Good Hygiene Practice for Food”. Meanwhile, sampling is conducted on targets of the Post-market Surveillance of Foods, as well as on products or raw materials that have been identified as of concern or high-risk by on-site inspections. The sample size is determined referring to the “Food Hygiene Inspection Items—Sample Size Table” announced by the Taiwan Food and Drug Administration, and each sample for testing animal drug items should be more than 150 g (edible portion). Through joint division of labor or each department’s own inspection, for products with drug residues exceeding the legal limits, the local health units will notify the traders to recall the products and remove them from the shelves, and deal with the cases in accordance with the law. Meanwhile, the health units also trace back to the upstream manufacturers and suppliers to achieve management at the source [43,46]. 

## 5. Conclusions

This study analyzes a wide range of meat products in Taiwan, including domestic and imported products sourced from supermarkets, traditional markets, and bulk stores. Past studies have been more limited in the scope of products and sources analyzed. This study comprehensively tests for a broad range of 21 different β-agonists, going beyond just ractopamine to provide a complete picture of the β-agonist contamination in beef and pork meat products. The study also assesses dietary exposure and risk across different age groups in Taiwan using estimated daily intake (EDI) and target hazard quotient (THQ) methods, presenting data on dietary risks rather than just residue detection. This study’s limitations include that we only evaluated samples of commercially available imported and domestic beef (including offal and fat) and pork (including offal and fat) products in post-market surveillance in New Taipei City, and the study does not take into account the dietary risk of other meat products. Moreover, the dietary intake acquired from the National Food Consumption Database was multiplied by the proportion of imported beef (96.1%) in Taiwan to estimate the actual intake volume of imported beef, which may not represent the actual intake of imported beef of each age group.

No β-agonists were detected in domestic beef, domestic pork, or imported pork sold in Taiwan during 2019–2023, while ractopamine residues were detected in imported beef. The maximum concentration of ractopamine detected in the samples was close to the MRL of ractopamine residue, at 10 μg/kg, according to the “Standards for veterinary drug residue limits in foods”; β-agonist residues in commercially available meat should be continuously monitored. The THQs for ractopamine in imported beef samples were all less than 1, indicating no health hazards at the current intake levels of each age group and the residue concentrations in commercially available beef.

## Figures and Tables

**Table 1:** Number of domestic and imported samples of commercially available beef and pork (including offal and fat), 2019–2023.

Year	Beef		Pork		Sample Size Subtotal
	Domestic	Imported	Domestic	Imported	
2019	11	18	95	1	125
2020	3 ^a^	25 ^b^	97 ^c^	21 ^d^	146
2021	16	127	583 ^e^	172 ^f^	898
2022	2	21	143	33	199
2023 ^h^	0	15	29 ^g^	3	47
Total	32	206	947	230	1415

^a^ One piece of beef tripe. ^b^ One piece of beef liver, three pieces of beef tripe, and one piece of beef heart. ^c^ Two pieces of pork liver and one piece of pork pancreas. ^d^ Three pieces of pork intestine. ^e^ Four pieces of pork liver, one piece of pork tripe, eight pieces of pork intestine, and one piece of pork heart. ^f^ Thirteen pieces of pork intestine. ^g^ One piece of pork liver and one piece of pork intestine. ^h^ The sample was collected from the Post-market Surveillance of Foods during the period from January to April 2023.

**Table 2:** Number and positive-for-beta-agonist residues of detected domestic and imported commercially available beef and pork (including offal and fat) samples, 2019–2023.

Year	Beef			Pork		
	Sample Size	Detected Samples		Sample Size	Detected Samples	
		Domestic	Imported ^a^		Domestic	Imported
2019	29	ND	5	96	ND	ND
2020	28	ND	8	118	ND	ND
2021	143	ND	26	755	ND	ND
2022	23	ND	2	176	ND	ND
2023 ^b^	15	ND	2	32	ND	ND
Total	238	ND	43	1177	ND	ND

Abbreviation: ND is not detected. ^a^ From the United States. ^b^ Sample was collected from the Post-market Surveillance of Foods during the period from January to April 2023.

**Table 3:** Concentrations of ractopamine residues and positivity rates of imported samples of commercially available beef, 2019–2023.

Year	Residual Concentration (μg/kg)	Mean ± SD (μg/kg)	Number of Detected Cases (Number of Imported Samples)	Positivity Rate ^a^ (%)
2019	2–6 ^b^	4.0 ± 01.4	5 (18)	27.8
2020	1–7 ^b^	4.1 ± 1.7	8 (25)	32.0
2021	1–7 ^b^	2.9 ± 1.4	26 (127)	20.5
2022	1–10 ^b^	5.5 ± 6.4	2 (21)	9.5
2023 ^c^	1–4 ^b^	2.5 ± 2.1	2 (15)	13.3
Total	1–10 ^b^	3.3 ± 1.9	43 (206)	20.9

^a^ Number of detected cases/Number of imported samples. ^b^ Minimum—Maximum. ^c^ Sample was collected from the Post-market Surveillance of Foods during the period from January to April 2023.

## Data Availability

The data used to support the findings of this study can be made available by the corresponding author upon request.

## Supplementary Materials

The following supporting information can be downloaded at https://www.mdpi.com/article/10.3390/foods12224052/s1, Table S1. The chromatographic and analytical conditions. Table S2. Average daily intake of meat or the 95th percentile intake of each age group in 2017–2020 statistics on daily meat intake in the Taiwan National Food Consumption Database [22]. Table S3. EDI and THQ of ractopamine in beef of each age group in terms of the average intake. Table S4. THQ and EDI of ractopamine in beef of each age group in terms of the 95th percentile intake.
