# Supplementary material for: Dietary Exposure and Risk Assessment of Beta-Agonist Residues in Commercial Beef and Pork in Taiwan
Source: Foods. 2023 Nov 7;12(22):4052. doi: 10.3390/foods12224052 (PMC10670263; doi:10.3390/foods12224052)
Supplement: Supplementary file 1 [file foods-12-04052-s001.zip › foods-2615654-supplementary.pdf]

Table S1. The chromatographic and analytical conditions.

Time (min)	Mobile phase A (%) 0.1% formic acid v/v	Mobile phase B (%) 0.1% formic acid: methanol v/v	Flow rate (mL/min)
0→0.5	98→98	2→2	0.3
0.5→1	98→90	2→10	0.3
1→5	90→80	10→20	0.3
5→8	80→70	20→30	0.3
8→10	70→60	30→40	0.3
10→11	60→60	40→40	0.3
11→12	60→10	40→90	0.3
12→15	10→10	90→90	0.3
15→19	10→10	90→90	0.3

Parameters: the Injection volume is 5 µL; drying gas temperature is maintained at 300°C; drying gas flow rate is set at 18 L/min; the nebulizer pressure is set at 35 psi; the capillary voltage is set to 4 kV; ion source temperature is maintained at 150°C.

Table S2. Average daily intake of meat or the 95th percentile intake of each age group in 2017–2020 statistics on daily meat intake in the Taiwan National Food Consumption Database [22].

Intake rate (g/day)		Age group (Body weight, kg)						
		0–3 (12.89)	3–6 (20.05)	6–12 (36.52)	12–16 (57.63)	16–18 (62.66)	19–65 (66.30)	> 65 (62.43)
Prok	Average ± SE	37.53 ± 1.97	52.79 ± 1.96	71.67 ± 1.95	77.12 ± 2.68	87.07 ± 4.56	74.18 ±1.36	63.90 ± 1.50
	95th percentile	106.99	141.87	191.55	188.13	217.67	196.44	167.90
Beef	Average ± SE	32.92 ± 3.33	49.79 ±5.23	89.95 ± 7.40	83.23 ±5.92	108.17 ± 11.70	89.44 ± 4.31	74.58 ±5.60
	95th percentile	80.62	152.96	279.07	211.18	335.11	241.52	188.38

Abbreviations: SE is the standard error.

Table S3. EDI and THQ of ractopamine in beef of each age group in terms of the average intake.

Concentration(μg/kg) \ Age(year)		0–3	3–6	6–12	12–16	16–18	19–65	> 65
Maximum value (20.0) [a]	EDI (μg/kg/day)	0.0491	0.0477	0.0473	0.0278	0.0332	0.0259	0.0230
	THQ	0.0491	0.0477	0.0473	0.0278	0.0332	0.0259	0.0230
MRL (10.0)[b]	EDI (μg/kg/day)	0.0245	0.0239	0.0237	0.0139	0.0166	0.0130	0.0115
	THQ	0.0245	0.0239	0.0237	0.0139	0.0166	0.0130	0.0115
Average (3.3)	EDI (μg/kg/day)	0.0081	0.0079	0.0078	0.0046	0.0055	0.0043	0.0038
	THQ	0.0081	0.0079	0.0078	0.0046	0.0055	0.0043	0.0038

Abbreviations: EDI is the estimated daily intake; THQ is the target hazard quotient; MRL is the maximum residue limit.

[a] Maximum concentration of ractopamine residue refers to the concentration of 20 μg/kg detected in beef at border inspections in 2021[13].

[b] [22].

Table S4. EDI and THQ ractopamine in beef of each age group in terms of the 95th percentile intake.

Concentration(μg/kg) \ Age(year)		0–3	3–6	6–12	12–16	16–18	19–65	> 65
Maximum value (20.0) [a]	EDI (μg/kg/day)	0.1202	0.1466	0.1469	0.0704	0.1028	0.0700	0.0580
	THQ	0.1202	0.1466	0.1469	0.0704	0.1028	0.0700	0.0580
MRL (10.0)[b]	EDI (μg/kg/day)	0.0601	0.0733	0.0734	0.0352	0.0514	0.0350	0.0290
	THQ	0.0601	0.0733	0.0734	0.0352	0.0514	0.0350	0.0290
Average (3.3)	EDI (μg/kg/day)	0.0198	0.0242	0.0242	0.0116	0.0170	0.0116	0.0096
	THQ	0.0198	0.0242	0.0242	0.0116	0.0170	0.0116	0.0096

Abbreviations: EDI is the estimated daily intake; THQ is the target hazard quotient; MRL is the maximum residue limit.

[a] Maximum concentration of ractopamine residue refers to the concentration of 20 μg/kg detected in beef at border inspections in 2021[13].

[b] [22].
